# Development of a Korean Liver Allocation System using Model for End Stage Liver Disease Scores: A Nationwide, Multicenter study

**DOI:** 10.1038/s41598-019-43965-2

**Published:** 2019-05-16

**Authors:** Juhan Lee, Jae Geun Lee, Inkyung Jung, Dong Jin Joo, Soon Il Kim, Myoung Soo Kim, Advisory Committee on Improving Liver Allocation, Choon Hyuck David Kwon, Dong-Sik Kim, Yang Won Nah, Hee-Jung Wang, Young Kyoung You, Hee Chul Yu, Kwang-Woong Lee, Dong Lak Choi, In Seok Choi, Shin Hwang

**Affiliations:** 1 Department of Surgery, Yonsei University College of Medicine, Seoul, Republic of Korea; 2 The Advisory Committee on Improving Liver Allocation, Seoul, Republic of Korea; 3 Department of Biostatistics and Medical Informatics, Yonsei University College of Medicine, Seoul, Republic of Korea; 4 Samsung Medical Center, Sungkyunkwan University School of Medicine, Seoul, Republic of Korea; 5 Korea University College of Medicine, Seoul, Republic of Korea; 6 Ulsan University Hospital, University of Ulsan College of Medicine, Ulsan, Republic of Korea; 7 Ajou University School of Medicine, Suwon, Republic of Korea; 8 Seoul St. Mary’s Hospital, College of Medicine, The Catholic University of Korea, Seoul, Republic of Korea; 9 Chonbuk National University College of Medicine, Jeonju, Republic of Korea; 10 Seoul National University College of Medicine, Seoul, Republic of Korea; 11 Daegu Catholic University School of Medicine, Daegu, Republic of Korea; 12 Konyang University Hospital, Konyang University College of Medicine, Daejeon, Republic of Korea; 13 Asan Medical Center, University of Ulsan College of Medicine, Seoul, Republic of Korea

**Keywords:** Hepatology, Outcomes research

## Abstract

The previous Korean liver allocation system was based on Child-Turcotte-Pugh scores, but increasing numbers of deceased donors created a pressing need to develop an equitable, objective allocation system based on model for end-stage liver disease scores (MELD scores). A nationwide, multicenter, retrospective cohort study of candidates registered for liver transplantation from January 2009 to December 2011 was conducted at 11 transplant centers. Classification and regression tree (CART) analysis was used to stratify MELD score ranges according to waitlist survival. Of the 2702 patients that registered for liver transplantation, 2248 chronic liver disease patients were eligible. CART analysis indicated several MELD scores significantly predicted waitlist survival. The 90-day waitlist survival rates of patients with MELD scores of 31–40, 21–30, and ≤20 were 16.2%, 64.1%, and 95.9%, respectively (*P* < 0.001). Furthermore, the 14-day waitlist survival rates of severely ill patients (MELD 31–40, n = 240) with MELD scores of 31–37 (n = 140) and 38–40 (n = 100) were 64% and 43.4%, respectively (*P* = 0.001). Among patients with MELD > 20, presence of HCC did not affect waitlist survival (*P* = 0.405). Considering the lack of donor organs and geographic disparities in Korea, we proposed the use of a national broader sharing of liver for the sickest patients (MELD ≥ 38) to reduce waitlist mortality. HCC patients with MELD ≤ 20 need additional MELD points to allow them equitable access to transplantation. Based on these results, the Korean Network for Organ Sharing implemented the MELD allocation system in 2016.

## Introduction

Liver transplantation is the treatment of choice for end-stage liver disease, fulminant hepatic failure, and for selected cases of hepatocellular carcinoma (HCC), but the demand for liver grafts vastly exceeds supply worldwide. In the West, efforts have focused on promoting deceased donor organ donation, and a model for end-stage liver disease score (MELD score) system has been implemented to enable equitable liver graft distribution^1–4^. On the other hand, in Asia the focus has been on living donor liver transplantation (LDLT)^5^, and the development of organ allocation system has been relatively neglected because deceased donation rates have been extremely low.

The previous Korean organ allocation system, which was introduced in 2000, was based on medical urgency and Child-Turcotte-Pugh scores (CTP scores). Although the shortcomings of the CTP score-based allocation system are well known^6^, they have not been of major concern in Korea due to the small number of deceased donor liver transplantation (DDLT) cases^7^. However, the number of deceased donors has increased since 2008, and there is now a growing need for an adequate, objective organ allocation system in Korea^8^.

The adoption of a MELD score-based system does not ensure equitable organ distribution^9^. In fact, MELD score-based systems have been continuously modified to enhance equity and efficiency^4,10,11^. Notably, the etiologies of liver diseases, medical environments, proportion of HCC, and donation rates are quite different in Asia and Western countries^5^. However, few studies have evaluated the feasibility of a MELD based allocation system in Korea^7^.

This study was undertaken to assess the feasibility of a MELD score-based allocation system using nationwide data. Based on this study, we sought to develop the Korean liver allocation system.

## Results

### Liver transplantation in Korea

**Figure 1:**
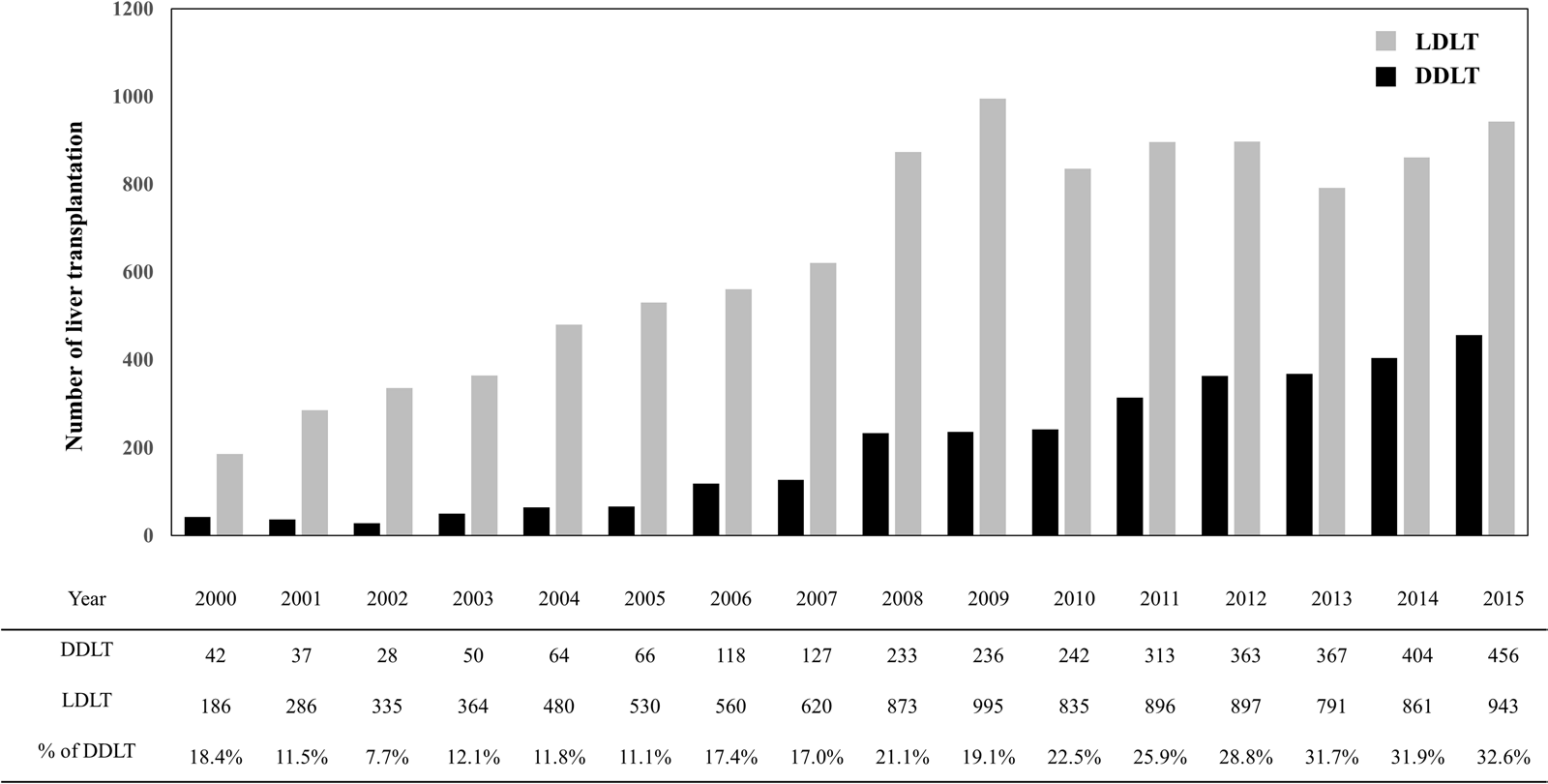
Liver transplantations in Korea.

The number of liver transplantations conducted annually in Korea reached a maximum of 1399 in 2015. LDLT accounts for the major proportion of liver transplantations conducted in Korea, and in 2015 the living donation rate was 18.73 per million population (PMP). However, numbers of LDLTs have remained static over the past few years despite the introduction of novel surgical techniques, whereas proportion of DDLT has increased steadily as a result of national efforts^8^. In 2015, DDLT accounted for 32.6% of liver transplantation cases (deceased donation rate 9.07 PMP, Fig. 1).

### Previous organ allocation system in Korea

In 2000, the Korean Network for Organ Sharing adopted the United Network for Organ Sharing classification for liver allocation, which is based on CTP score and status^12^. Highest priority (status 1) was given to patients with fulminant hepatic failure or acute graft failure within 7 days after liver transplantation. The next level of urgency was status 2A, which was defined as patients with chronic liver disease who had a CTP score of ≥10 and meet at least one of the other medical criteria (life-threatening variceal bleeding, refractory ascites/hydrothorax, hepatic encephalopathy, or hepatorenal syndrome), who were hospitalized in an intensive care unit with a life expectancy of <7 days without a liver transplant. Status 2B was defined as patients with chronic liver disease who had a CTP score of ≥10, or a CTP score of ≥7 and meet at least one of the medical criteria (life-threatening variceal bleeding, refractory ascites/hydrothorax, or spontaneous bacterial peritonitis). Patients with stage T1 and T2 HCC were allocated status 2B. Status 3 was established in patients who had a CTP score of ≥7 without meeting status 2B criteria.

**Figure 2:**
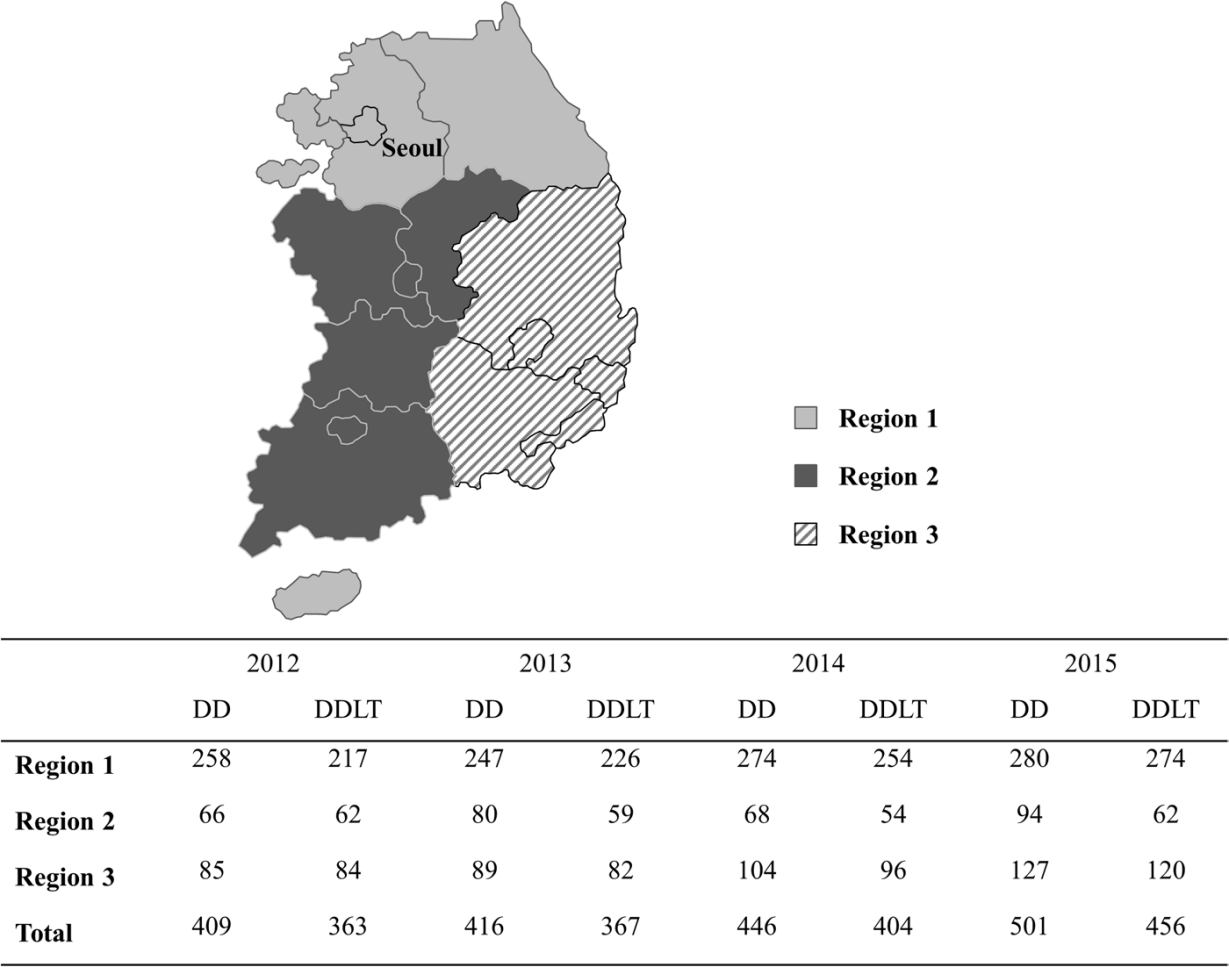
Number of deceased donors and liver transplantation in Korea by geographic regions.

Organ sharing areas in Korea consist of regional and national tiers since geographic distances are relatively short. The distribution policy based on arbitrary geographic boundaries divides Korea into three regions (Fig. 2). Region 1 includes the capital city (Seoul) and covers 30% of the land mass but accounts for 54% of the population and 58% of transplant centers. This heterogeneity between regions is the result of regional organ donation and transplant activity differences. To reduce waiting list mortality for severely ill patients, regardless of region, the previous Korean allocation system offers national priority to Status 1 and 2A patients for 2 weeks from the registration.

Between 2011 and 2015, 57.2% of DDLTs (1088/1903) were performed in the urgent patients (8.1% in status 1, 49.1% in status 2A). Status 2B patients were given the opportunity only if there were no status 1 or 2A patients on the waiting list^13^.

### Patient characteristics

A total of 2702 patients were placed on the liver transplantation waiting lists from January 2009 to December 2011 at the 11 transplantation centers. The exclusion criteria were pediatric candidates, fulminant hepatic failure, and unknown liver disease. Baseline characteristics obtained at registration are provided in Table 1. Overall, mean age at registration was 53.5 years. The majority of 2248 patients were male (73.4%) and most (71.0%) were hepatitis B virus (HBV) carriers. Based on the CTP scoring system of liver functions, 16.6% were class A, 41.7% were class B, and 41.7% were class C. Their mean laboratory MELD score was 17.1, mean follow-up duration was 362 days, and 1071 (47.6%) had HCC. Compared to non-HCC patients, HCC patients were older (*P* < 0.001) and more likely to be male (*P* < 0.001). In addition, HCC patients were more likely to have HBV infection, but less likely to have alcoholic cirrhosis (*P* < 0.001). HCC patients tended to retain liver function at registration, and thus, had lower CTP and MELD scores than non-HCC patients (*P* < 0.001). Of the 1395 status 2B patients, 122 non-HCC patients (18.3%) had a MELD score of >30, whereas only 14 HCC patients (1.9%) had a MELD score of >30.

**Table 1:** Patient characteristics.

	Overall patients	Patients without HCC (n = 1177)	Patients with HCC (n = 1071)	*P*-value
Age at listing (years)	53.5 ± 9.1	51.7 ± 9.6	55.5 ± 7.9	<0.001
Male, n (%)	1650 (73.4%)	823 (69.9%)	827 (77.2%)	<0.001
Blood type, n (%)				0.971
A	776 (34.5%)	403 (34.2%)	373 (34.9%)	
B	654 (29.1%)	344 (29.2%)	310 (28.9%)	
AB	250 (11.1%)	134 (11.4%)	116 (10.8%)	
O	568 (25.3%)	296 (25.2%)	272 (25.4%)	
Body mass index (kg/m^2^)	24.1 ± 3.5	24.0 ± 3.7	24.2 ± 3.3	0.12
Original liver disease, n (%)				<0.001
Hepatitis B virus	1595 (71.0%)	707 (60.1%)	888 (82.9%)	
Hepatitis C virus	200 (8.9%)	93 (7.9%)	107 (10.0%)	
Alcoholic	360 (16.0%)	304 (25.8%)	56 (5.2%)	
Cholestatic	40 (1.8%)	37 (3.1%)	3 (0.3%)	
Others	53 (2.3%)	36 (3.1%)	17 (1.6%)	
CTP class, n (%)				<0.001
A	374 (16.6%)	79 (6.7%)	295 (27.5%)	
B	938 (41.7%)	397 (33.7%)	541 (50.6%)	
C	936 (41.7%)	701 (59.6%)	235 (21.9%)	
MELD score	17.1 ± 8.9	20.5 ± 9.2	13.4 ± 6.6	<0.001
Status*, n (%)				<0.001
2A	159 (7.1%)	122 (10.4%)	37 (3.5%)	
2B	1395 (62.1%)	665 (56.5%)	730 (68.2%)	
3	694 (30.8%)	390 (33.1%)	304 (28.3%)	

*Status based on previous Korean liver allocation system.

MELD score distribution according to status are shown in Table 2. In the CTP score-based system, there was a broad range of liver disease severity in the same status. In addition, some status 2B candidates with high MELD score received lower priority than status 2A candidates with lower MELD score. In our cohort, 68 status 2A patients (42.8%) had a MELD score of ≤30, whereas 136 status 2B patients (10.0%) had a MELD score of >30.

**Table 2:** MELD score distribution in status 2A and status 2B patients.

	Status 2A (n = 159)	Status 2B (n = 1395)
MELD ≤ 20	22 (13.8%)	968 (69.4%)
MELD 21–30	46 (28.9%)	291 (20.9%)
MELD 31–37	47 (29.6%)	85 (6.1%)
MELD 38–40	44 (27.7%)	51 (3.7%)

### Survival analysis for stratification

**Figure 3:**
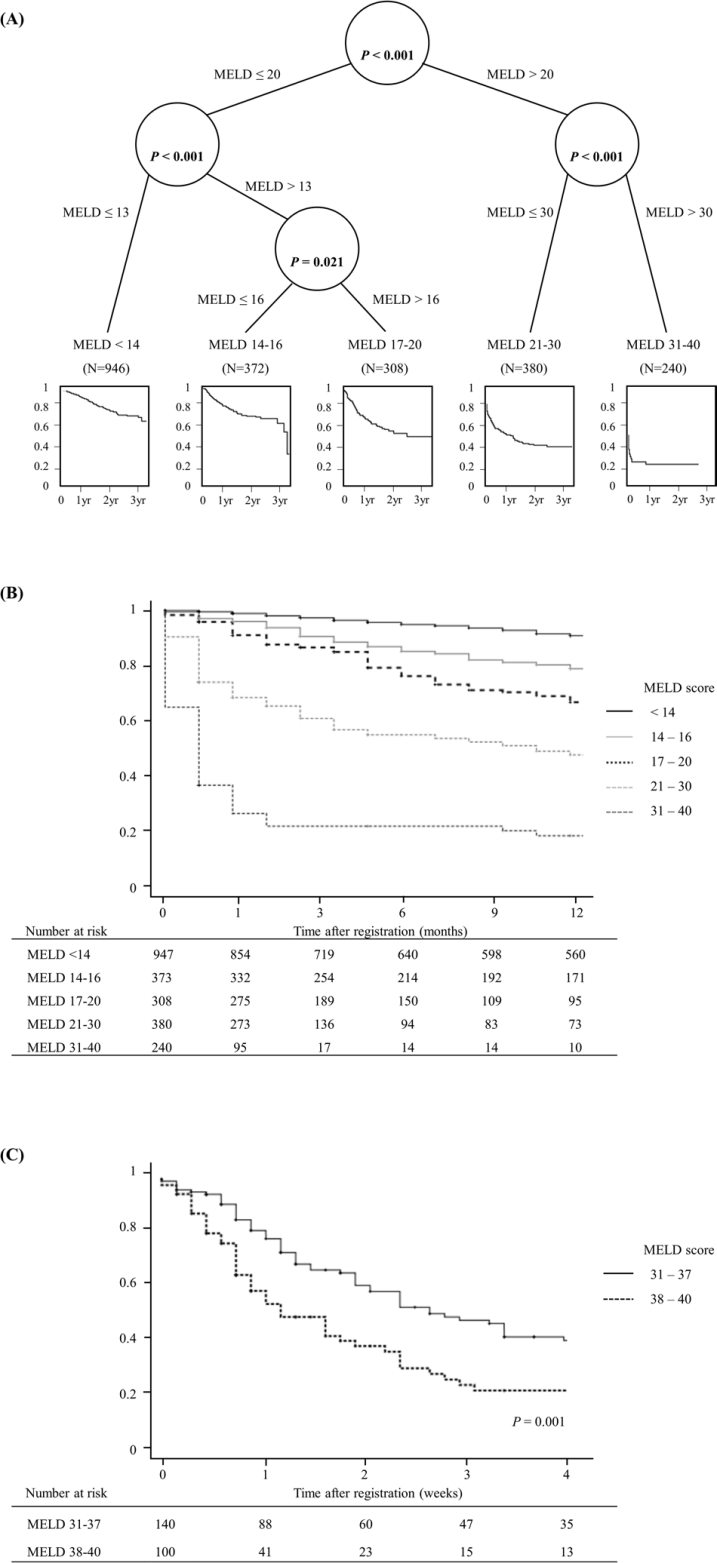
Waiting list survival. (**A**) Classification and regression tree; (**B**) Waiting list survival rate by MELD score; (**C**) Waiting list survival rate for severely ill patients.

Using classification and regression tree (CART) analysis, we successively partitioned chronic liver disease patients into subgroups (Fig. 3A). CART analysis indicated that a MELD score cut-off of 20 was a primary determinant of waitlist survival. In patients with a MELD score of >20, the next most important cut-off score was 30. Ninety-day waitlist survival rates were 16.2% and 64.1% for patients with MELD scores 31–40 or 21–30, respectively. Patients with a MELD score of ≤20 had a 90-day waitlist survival rate of 95.9% (Fig. 3B).

### Severely ill patients (MELD score >30)

Of the 2248 study subjects, 240 (10.7%) had a MELD score of >30. However, short-term waitlist survival for these patients was not homogeneous, and thus, these 240 patients were partitioned into two subgroups by CART analysis. Patients with MELD score of <38 had a significantly lower waitlist removal rate than those with a MELD score of 38–40. For patients with MELD scores of 31–37 or 38–40, 14-day waitlist survival rates were 64% and 43.4%, respectively (*P* = 0.001, Fig. 3C).

### MELD exception for hepatocellular carcinoma

**Figure 4:**
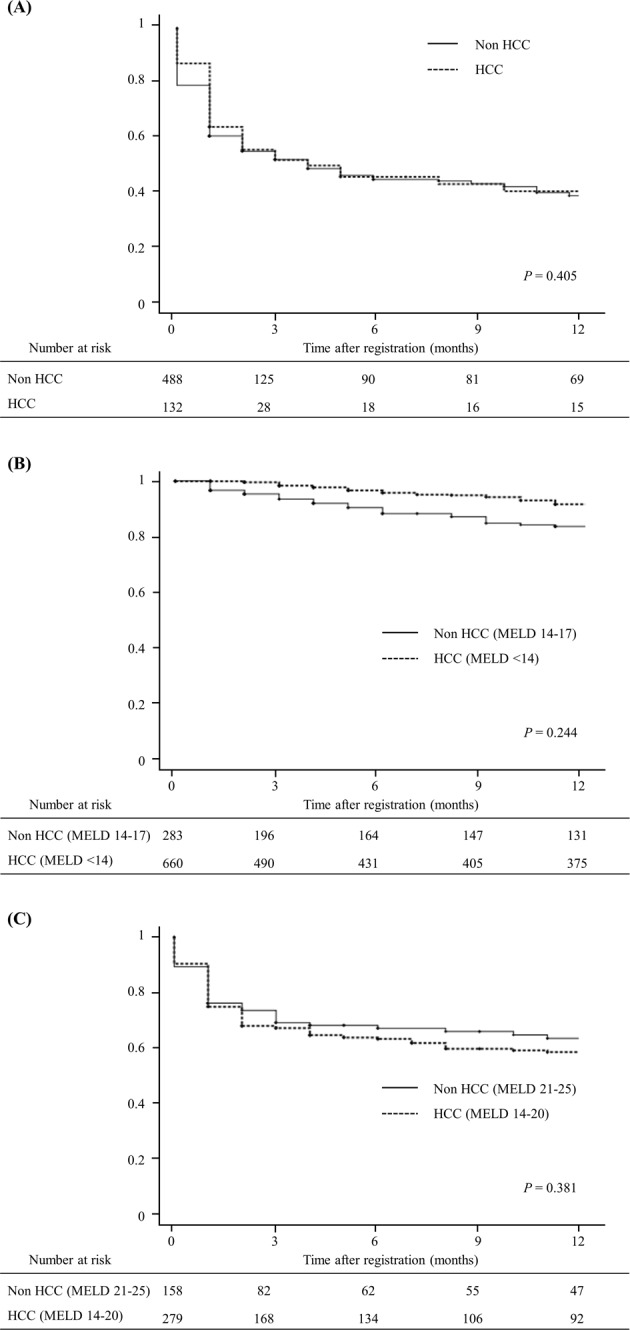
Waiting list survival rates between HCC and non-HCC patients by MELD score. (**A**) Waiting list survival in HCC patients with MELD score of >20; (**B**) Waiting list survival in HCC patients with MELD score of <14; (**C**) Waiting list survival in HCC patients with MELD score of 14–20.

In patients with a MELD score of >20, the presence of HCC did not affect waitlist survival (Fig. 4A). HCC patients with a MELD score of ≤20 were compared to non-HCC patients with certain MELD ranges. The MELD score ranges of non-HCC patients were chosen using CART analysis. The 90-day outcomes were similar for patients with HCC and a MELD score of <14 and patients without HCC and a MELD score of 14–17 (Fig. 4B), and waitlist survival rates were similar for patients with HCC and a MELD score of 14–20 and patients without HCC and a MELD score of 21–25 (Fig. 4C).

## Discussion

The goal of organ allocation is to distribute organs in a fair and ethical fashion based on medical judgment. Because the number of DDLT procedures is increasing in Korea, there is a pressing need to reorganize the liver allocation system. Hence, we conducted this multicenter study to assess the feasibility of MELD score and to develop Korean liver allocation system based on MELD score.

The previous liver allocation system used in Korea prioritizes waitlist candidates based on medical urgency and CTP score, but although the CTP scoring system can be used to stratify disease severity intuitively, its discriminatory ability is limited^6,7^. Hence, non-medical factors, such as, waiting time and center incentive have become the primary means of prioritizing patients within each status designation^7,14^. Furthermore, some status 2B candidates with high MELD score received lower priority than status 2A candidates with lower MELD score. If liver grafts are allocated according to the MELD score, it is expected to reduce waiting list mortality.

The MELD score is a scoring system for assessing the severity of chronic liver disease and provide a continuous, transparent, and objective measure of 90-day waitlist mortality^15,16^. Hence, offering donor livers in descending MELD score order can be a simple and reliable means of liver allocation for chronic liver disease candidates. However, in the real world, geographic boundaries and supply-demand ratios should also be considered. Although not intended, MELD score-based allocation systems caused significant geographic disparities^17^. In addition, chronic liver disease patients with high MELD score had similar or even higher waiting list mortality than fulminant hepatic failure patients (status 1) who benefit from broader sharing of livers^18^. To address this issue, several countries implemented broader sharing of livers not only for the fulminant hepatic failure patients but also for the high MELD patients^4,10,11,19^. For such systems, arbitrary cut-off scores are chosen based on waitlist mortalities on a countrywide basis.

In the present study, CART analysis was used to identify arbitrary MELD score ranges that reflect meaningful waitlist survival differences in Korea. According to CART analysis, 90-day waitlist survival rates were found to be significantly different for MELD score ranges of 31–40, 21–30, and ≤20. Subgroup analysis showed the 14-day waitlist survival rate was 43.4% for patients with a MELD score of 38–40 and 64% for those with a MELD score of 31–37. Accordingly, we proposed national broader sharing of liver allografts for the patients with MELD score of 38 or higher in order to reduce waitlist deaths (Table 3).

**Table 3:** Staged broader sharing model based on MELD score.

US Liver allocation scheme (UNOS)	Korean Liver allocation model (KONOS)
Combined local and regional status 1A/1B	Combined regional and national status 1
Local/regional MELD score 35–40 (offers made locally then regionally for each MELD score)	Regional/national MELD score 38–40 (offers made regionally then nationally for each MELD score)
Local MELD score 15–34	Regional MELD score 31–37
Regional MELD score 15–34	National MELD score 31–37
National status 1A/1B	Regional MELD score 21–30
National MELD score ≥15	National MELD score 21–30
Local MELD score <15	Regional MELD score ≤20
Regional MELD score <15	National MELD score ≤20
National MELD score <15	

MELD score does not allow fair access to liver transplantation for HCC patients because in these patients’ liver function is often preserved. For this reason, in many countries, selected HCC patients are awarded an arbitrary MELD score in an attempt to estimate the risk of HCC progression beyond Milan criteria^3,4,16^. However, the risk of waitlist removal due to HCC progression was much less than originally estimated. Despite constant adjustments, recent studies have demonstrated HCC patients have easier access to transplantation than non-HCC patients^20–22^. Furthermore, in Korea where LDLTs are performed in HCC patients beyond Milan criteria, equitable selection is more complicated than in other countries. Hence, we compared waitlist survival rather than HCC progression to select HCC candidates warranting additional MELD points.

Prioritization within HCC candidates is another important issue. If all HCC candidates with a MELD score of ≤20 were awarded the same exceptional points, non-medical parameters would dominate allocations. Several models based on MELD score and tumor characteristics have been proposed to achieve more equitable distributions between HCC and non-HCC patients^22–25^. However, these models have not been well validated. Moreover, the use of tumor aggressiveness to determine priorities among HCC patients is likely to increase the risk of posttransplant HCC recurrence. On the other hand, laboratory MELD scores are known well predict HCC patient dropout from waitlists^26^. Hence, we developed an evidence-based model according to the waitlist survival rates of HCC and non-HCC patients in Korea. HCC MELD exception was determined through advisory meetings based on our nationwide data^27^. The KONOS provides additional points for patients with T2 HCC based on laboratory MELD score (HCC patients with MELD score of <14 receive an additional 4 points and those with MELD score between 14 and 20 receive an additional 5 points). With the introduction of the MELD system in the current organ donation rate, we expect that HCC patients with preserved liver function are less likely to receive DDLT. In order to resolve the disparity in access to DDLT, efforts should be made not only to improve the allocation system but also to increase the number of deceased donors.

In the setting of organ scarcity, not only urgency but also utility or transplant outcome should be considered. In fact, reports issued in several countries have described poor post-transplant outcomes after adoption of a MELD system^28^. However, relation between MELD scores and survival following transplantation remains controversial^29,30^. In addition, the high proportion of LDLTs conducted in Korea makes it much more difficult to predict the consequences of adopting a MELD system. Therefore, future studies are needed to assess the impact of MELD system on transplant outcomes.

The Korean liver allocation system is not perfect and there is room for improvement. First, we have not yet set exceptional points for other indications other than HCC. Instead, if a candidate’s transplant physician believes that a candidate’s MELD score does not appropriately reflect the candidate’s medical urgency, the physician may request an exceptional priority to the KONOS. Second, we do not have specific regulation for patients using vitamin K-antagonists. The international normalized ratio, which has the largest weight in the MELD score, is significantly affected by vitamin K-antagonists. The Eurotransplant has a specific regulation for patients using vitamin K-antagonists considering its importance^3^. Continuous efforts to improve allocation system will be needed in the future.

Despite its retrospective design and short follow-up, this is the first study to investigate the availability of the MELD score-based allocation system using national cohort data in Asia. Although MELD scores may accurately predict waitlist mortality, they do not ensure equitable organ distribution^9^. Medical environments, organ donation rates, proportion of LDLT, and regional distributions differ markedly between countries. Hence, thorough verification is required before adopting a new allocation system. We believe the present study may provide valuable basis for developing MELD score-based allocation system in other countries.

In conclusion, the MELD score system adequately stratified the short-term mortality rates of chronic liver disease patients on Korean waitlists. Regarding donor organ shortages and geographic disparities, we proposed the broader sharing of livers for the sickest patients (MELD score of ≥38) to reduce waitlist mortality. Our findings demonstrated that HCC patients with a MELD score of ≤20 need additional MELD points to allow them equitable access to transplantation. The MELD score allocation system was finally implemented in Korea in 2016 after a series of advisory committee meetings based on the results of this study.

## Materials and Methods

### Subjects

**Figure 5:**
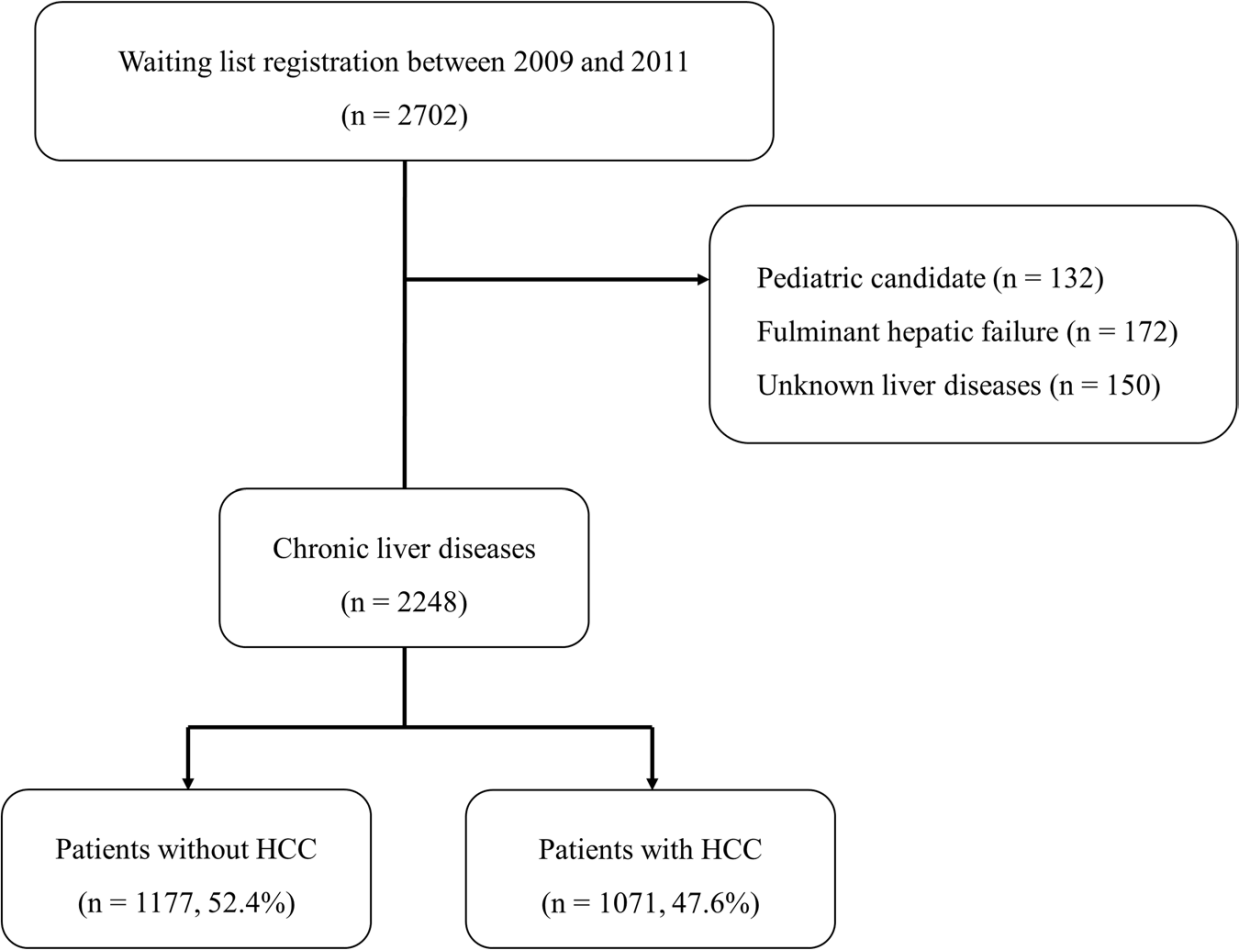
Study design.

A nationwide, multicenter, retrospective cohort study was performed on 2702 candidates registered for liver transplantation from January 2009 to December 2011 at 11 liver transplantation centers, which conducted 83.1% of Korean liver transplantations (1047/1260) in 2012. The exclusion criteria applied were pediatric cases, fulminant hepatic failure, and unknown liver disease. Patients were followed until 31 March 2013 to ensure that all were followed for at least one-year. These patients were divided into two groups according to the presence of HCC (Fig. 5). All donor surgeries were performed with the consent of the donor and approval from the Korean Network Organ Sharing. No allografts (organs and tissue) obtained from prisoners were used.

### Parameters

Baseline information, such as, age, sex, body mass index, original liver disease, CTP score, presence of HCC, and blood type were obtained at time of registration.

MELD scores were calculated at the time of registration using the following equation:

$$\begin{array}{rcl}{\rm{MELD}}\,{\rm{score}} & = & (0.957\,{\mathrm{log}}_{{\rm{e}}}\,{\rm{creatinine}}+0.378\,{\mathrm{log}}_{{\rm{e}}}\,{\rm{bilirubin}}\\  &  & +\,1.12\,{\mathrm{log}}_{{\rm{e}}}\,{\rm{international}}\,{\rm{normalized}}\,{\rm{ratio}}+0.643)\ast 10.\end{array}$$

For this calculation, laboratory values of <1.0 were set at 1.0. The upper limit of serum creatinine was set at 4.0 mg/dL, and MELD scores were capped at 40. For patients who received two or more dialysis within the last week, or 24 hours of continuous veno-venous hemodialysis, the creatinine value were set to 4.0 mg/dL.

### Outcomes

The primary outcome was waitlist survival. Candidates removed from the waitlist because of clinical deterioration were considered to be equivalent to candidates that succumbed to chronic liver disease because clinical deterioration in such a situation is almost uniformly fatal without transplantation. These definitions are consistent with those used in previous studies^18^. For severely ill patients, we compared 14-day waitlist survival.

Waiting time was defined as time elapsed between registration and date of removal from the waiting list. Patients were censored if they were alive at last follow-up and on a waiting list or had been removed due to improvement or transplantation.

### Statistical analysis

Demographic information was summarized using frequencies (percentages) or means ± standard deviations. The Chi-square test with Fisher’s exact test was used to compare categorical variables and the student’s *t*-test was used to compare continuous variables. Survival and freedom from events were estimated using the Kaplan-Meier method and compared using the log-rank test.

CART analysis is a tree-based classification and prediction method that utilizes recursive partitioning to split records into segments with similar output field values. At each split, two child nodes are generated using a selected best predictor, at the next split, each child node may become a parent node and generate two child nodes. A unified framework was applied for recursive partitioning to avoid overfitting and variable selection problems^31^.

We performed CART analysis to identify MELD scores where the 90-day waitlist survival varies greatly. In severely ill patients, we compared waitlist survival within 14-day of listing^18^. Waitlist survivals were analyzed with respect to MELD scores and the presence of HCC in order to identify HCC candidates requiring additional MELD points. MELD score ranges were identified by CART analysis. Survival curves were compared using the log-rank test to identify MELD score adjustments for HCC patients that equalized waitlist outcomes for HCC and non-HCC patients.

The statistical analysis was performed using R (version 3.1.1; The R Foundation for Statistical Computing, Vienna, Austria), and *P*-values va0.05 were considered statistically significant.

### Ethical approval

All liver transplants were performed after obtaining informed consent at the 11 participating centers. The study was conducted according to the tenets of the Declaration of Helsinki and was approved by the Institutional Review Boards of Severance Hospital (2012-1142-001). Signed patient informed consent was waived per the IRB approval, since the study was a retrospective analysis.

## Data Availability

The datasets generated during and/or analyzed during the current study are available from the corresponding author on reasonable request.
